# Antifungal activity, mechanical properties, and accuracy of three-dimensionally printed denture base with microencapsulated phytochemicals on varying post-polymerization time

**DOI:** 10.1186/s12903-022-02654-9

**Published:** 2022-12-15

**Authors:** Ye-Hyeon Jo, Won-Jun Lee, Ji-Hyun Lee, Hyung-In Yoon

**Affiliations:** 1 Dental Research Institute, Seoul National University School of Dentistry, Seoul, Republic of Korea; 2 Department of Prosthodontics, School of Dentistry and Dental Research Institute, Seoul National University, 101, Daehak-ro, Jongno-gu, Seoul, 03080 Republic of Korea

**Keywords:** 3D printing, Denture base, Phytoncide, Microcapsule, Antifungal activity, Post-polymerization time

## Abstract

**Background:**

Studies on the antifungal activity, flexural strength, Vickers hardness, and intaglio surface trueness of three-dimensionally printed (3DP) denture bases with microencapsulated phytochemicals with respect to changes in post-polymerization time (PPT) are lacking.

**Methods:**

Specimens of various shapes and dimensions were fabricated with a 3DP denture base resin mixed with 5 wt% phytoncide-filled microcapsules. Each specimen was subjected to different PPT protocols of 5, 10, 20, and 30 min. Specimens without microcapsules with 5-min PPT were used as the negative control group. Cell colonies were counted to evaluate antifungal activity. Three-point bending and Vickers hardness tests were performed to measure the flexural strengths and hardness of the specimens. Fourier-transform infrared spectrometry was used to inspect the degree of conversion (DC). The intaglio surface trueness was measured using root-mean-square estimates calculated by superimposition analysis. A non-parametric Kruskal–Wallis test or one-way analysis of variance was performed (α = 0.05).

**Results:**

The specimens with microcapsules and 10-min PPT showed the highest antifungal activity among the tested groups. Compared with the positive control group (5-min PPT), the specimens with PPTs of 10 min or longer showed significantly higher mean flexural strength, higher DC, greater hardness, and better trueness (all, *P* < 0.05). Except for the difference in antifungal activity, no statistically significant differences were detected between the specimens subjected to 10-, 20-, and 30-min PPT.

**Conclusion:**

The 3DP denture base filled with microencapsulated phytoncide showed different antifungal activity and physical properties on changing PPT. The 3DP denture base containing phytoncide-filled microcapsules at 5 wt% concentration and subjected to 10-min PPT exhibited sufficient antifungal activity as well as mechanical properties and accuracy within clinical acceptance.

## Introduction

Polymethyl methacrylate (PMMA) resin has been used in the fabrication of removable denture prostheses for a long time [1–3]. It has several advantages, such as favorable esthetics, biocompatibility, low cost, and simple processing [1, 4]. However, fracture of the acrylic resin is frequently reported in 57–64% of all cases of clinical failures of removable denture prostheses [5, 6]. Furthermore, the conventional pack and press technique of denture fabrication has been reported to be susceptible to dimensional change owing to polymerization shrinkage [7, 8]. With the progress of computer-aided design and computer-aided manufacturing (CAD-CAM) technology, additive manufacturing methods, also known as three-dimensional (3D) printing, have been applied to the workflow of fabricating denture prostheses [9, 10]. 3D-printed (3DP) denture bases can be manufactured with unlimited shape reproduction, sufficient precision, and physical properties using a photo-polymerizable acrylic resin [9, 10], which simplifies the workflow of removable dentures [10]. Recently, the clinical applicability of 3DP denture bases has been substantiated by several researchers. Denture bases for edentulous arches fabricated using digital light processing (DLP) showed favorable tissue surface adaptation and dimensional accuracy, which was similar to or better than that of bases manufactured by 5-axis milling [11, 12]. A clinical study by Yoon et al. revealed that denture bases produced via 3D printing were more advantageous than those produced via milling in terms of tissue surface adaptation due to better reproduction of the complex contour of the edentulous ridge [13]. Furthermore, the adaptation of the denture base to the tissue surface can be improved by optimizing the build angle during the DLP-based workflow [14]. However, regardless of the manufacturing workflow, the acrylic resin material used for denture bases has an inherent drawback, as the intaglio surface could be a potential reservoir of oral microorganisms, such as bacteria or fungi, due to surface roughness, geometric irregularities, and porosity [15–17].

Denture stomatitis is an inflammatory fungal infection that affects approximately 50–75% of otherwise healthy denture wearers and is the most common form of oral candidiasis [18–20]. Apart from antifungal treatment, conditioning of the abused tissue, cleansing of the affected mucosa or denture surface, and fabrication of new dentures are the treatment of choice. Due to concerns regarding systemic adverse reactions caused by oral antifungal drugs [21, 22], alternative approaches, such as photodynamic treatment [23] or adjunct physical cleansing, have been recently used to effectively treat denture stomatitis [24]. In addition, imparting antibacterial activity to the denture material by directly coating or adding antibacterial compounds has also been proposed [25–28]. In particular, a novel additive manufacturing technique to directly add substances with antibacterial properties to the denture material has been suggested. The substances selected for this purpose were certain oxides or chemical compounds and phytochemicals, which are naturally derived substances with antimicrobial activities [29–31]. The antifungal properties of phytochemicals, such as phytoncide oil, have been previously reported [32, 33]. The phytoncide could reduce optical density and viability of fungal cells as well as increase the number of morphologically atypical cells [34]. Recent studies reported the development of a denture base with antifungal properties by incorporating phytoncide into PMMA resin [35, 36]. Furthermore, microencapsulation of phytoncide oil extract was recommended to achieve continuous effect of its antimicrobial activity [30, 36].

A novel method using microencapsulation and additive manufacturing has been recently introduced to incorporate the antifungal activity of phytoncide extract into the acrylic resin for 3DP denture bases and maintain its effectiveness for a certain period without compromise [31]. 3DP denture bases with phytoncide-filled microcapsules at concentrations ranging from 6 to 8 wt% showed sufficient antifungal effects on fungal cell colonies [31]. However, they showed mediocre mechanical properties (flexural strength or hardness), which were barely comparable with those of bases without microcapsules, and exhibited lesser dimensional accuracy, which was clinically acceptable [37]. Since denture bases should withstand masticatory forces, resist surface wear, and guarantee intimate tissue surface adaptation, the physical properties and dimensional accuracy of the 3DP denture base with phytoncide-filled microcapsules must be improved further. Among the various parameters used for 3D printing, the light exposure time during the post-polymerization has been reported to affect the accuracy and strength of 3DP resin materials [38–40]. The dimensional accuracy (trueness and precision), degree of conversion, and mechanical properties (flexural strength and Vickers hardness) of resin-based 3DP objects have been significantly improved by changing the post-polymerization time (PPT) [38–40].

To the best of our knowledge, there has been no research on the effect of PPT on the antifungal activity, physical properties, and accuracy of 3DP denture base resins containing phytochemical-filled microcapsules. Therefore, this in-vitro study aimed to evaluate 3DP denture bases with microencapsulated phytochemical (phytoncide oil extract) in terms of antifungal activity, flexural strength, Vickers hardness, and intaglio surface trueness with respect to changes in PPT (5, 10, 20, and 30 min). The null hypothesis was that the antifungal activity, flexural strength, Vickers hardness, and intaglio surface trueness of 3DP denture bases filled with microencapsulated phytoncide would not be affected by the changes in PPT.

## Methods

A disc-shaped specimen with a diameter of 15 mm and thickness of 5 mm was virtually designed with universal CAD software (TinkerCAD, Autodesk, Quebec) to test the antifungal activity, Vickers hardness, and degree of conversion. A bar-shaped specimen (65 × 10 × 3.3 ± 0.2 mm) was designed to measure the flexural strength of the printed denture base materials in accordance with ISO 20975-1:2013 [41]. A maxillary denture base-shaped specimen was designed to evaluate the trueness of the intaglio surface after 3D printing. An edentulous maxillary dentiform with soft gingiva (EDE1001-UL-UP-DPM, Nissin, Japan) was digitized to obtain maxillary full-arch scan data (i500; Medit, Korea). A maxillary complete denture base (reduced to 50% of its size) was virtually designed using dental CAD software (3Shape Dental Designer; 3Shape, Danmark) and saved in a standard tessellation language (.stl) format as reference CAD data.

A 3D-printable resin (NextDent Denture 3D+, Vertex Dental BV, Soesterberg, Netherlands), designed for the fabrication of removable denture bases, was used in this study. Phytoncide oil was extracted from *Pinus Densiflora* (Cleandiox. Ltd., Korea) and microencapsulated to prepare functional microcapsules with antifungal activity [31]. Detailed process of microencapsulation was previously reported in the study by Jeon et al. [31]. Briefly, the phytoncide oil and the styrene-maleic anhydride copolymer solution was mixed to form droplets. Melamine/urea/formaldehyde was then dissolved in the mixture to form a wall of the microcapsule. After aging for cohesion, the microcapsules were sieved and dried. The phytoncide-filled microcapsules were carefully mixed with the 3D-printable denture base resin at a concentration of 5 wt% [31, 37]. A dispersant (DISPERBYK-111, BYK, Germany) was added at 20 wt% of the mass (g) of the phytoncide-filled microcapsules to the mixture for uniform dispersion. All 3DP denture base resin specimens with various shapes and dimensions were produced using the microcapsule-resin mixture and a DLP-based 3D printer (MAX UV, ASIGA, Australia) with a wavelength of 385 nm. The layer thickness and build orientation for each 3D printing were set to 50 μm and 0°, respectively, and horizontally positioned to build the platform (Asiga Composer 1.2.12, Asiga, Australia). Specimens of 3DP denture base resin with no microcapsules and dispersant agent were also manufactured according to the abovementioned protocols and parameters as the control group. All specimens were cleaned using ethanol in a washing machine as per the manufacturer’s instructions (Form wash, Formlabs, Somerville, USA) for 10 min. The support structures were carefully removed, and no further adjustments were made to the specimen surfaces.

**Fig. 1:**
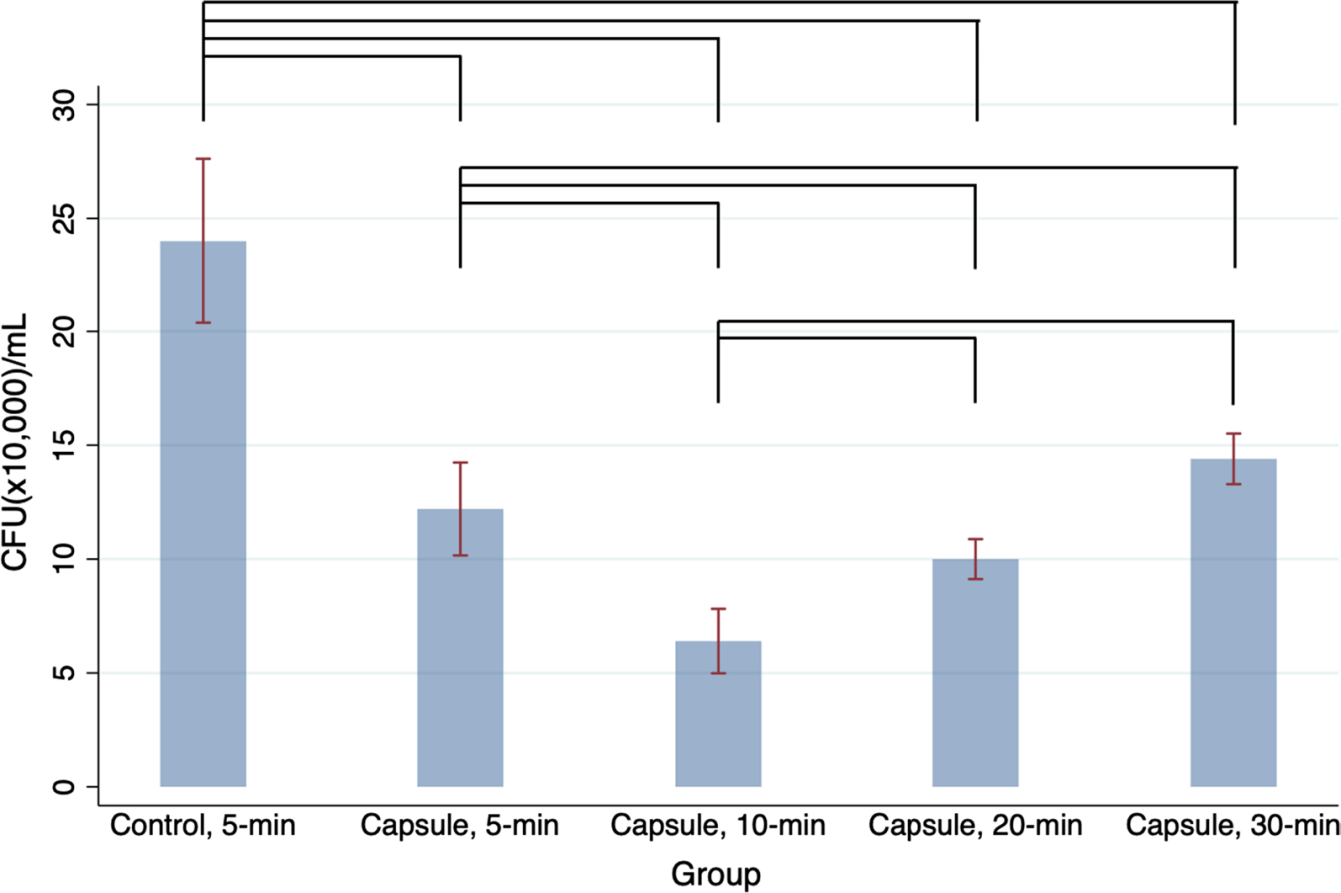
Colony-forming units per milliliter (CFU/mL) of *Candida albicans* detached from 3D-printed denture base resin discs and subjected to different post-polymerization time (PPT) protocols: 5-min, 10-min, 20-min, and 30-min. Control: 3D-printed denture base resin discs without microcapsules; Capsule: 3D-printed denture base resin discs with 5 wt% phytoncide-filled microcapsules. Significant differences between the groups are marked with black lines (*P* < 0.05)

After cleansing and removing the support structure, the disc-shaped 3DP denture resin specimens (Fig. 1) underwent post-polymerization with a curing unit (Cure M U102H, Graphy, Seoul, Korea). The 5-min PPT, a default protocol recommended by the manufacturer, was applied to the 3DP denture resin discs with phytoncide-filled microcapsules for the control group (positive control). For the experimental groups, 3DP denture resin discs with microcapsules subjected to three different PPTs (10-, 20-, and 30-min) were evaluated. As a negative control, the 3DP denture resin discs without microcapsules subjected to 5-min PPT (default protocol) were also tested. After post-processing as recommended by the manufacturers, the as-printed surface of each 3DP denture resin specimen, opposing the attached area of the support structures, was used as a testing surface for further analysis.

*Candida albicans* (ATCC 10,231, *C. albicans*), provided by the Korean Collection for Oral Microorganisms, was cultured in Sabouraud dextrose (SD) broth at 37 °C and used to assess the antifungal activity of the 3DP denture resin discs with phytoncide-filled microcapsules. The 3DP discs with and without microcapsules (n = 5 each), subjected to different PPT protocols, were placed at the bottom of a 12-well plate. To each well, 2 mL of *C. albicans* suspension (optical density: 0.02, equivalent to 2 × 10^4^ microbial cells per mL) was added and incubated for 24 h at 37 °C. The discs were rinsed with Dulbecco’s phosphate-buffered saline (DPBS) and transferred to tubes filled with 5 mL of SD broth. Each disc was ultrasonicated at 40 kHz for 5 min to detach the microbial cells from its surface (NXPC-B5020SB, Kodo, Korea) [42]. The absorbance value of the fungal solution (200 µL) was measured using a microplate reader (Epoch2, BioTek, USA) at a wavelength of 600 nm. Subsequently, 10 µL of the diluted fungal solution was seeded onto an SD agar plate and incubated for 24 h at 37 °C. For the SEM analysis, the discs were cleaned with DPBS and fixed in 2.5% (v/v) glutaraldehyde (Sigma-Aldrich, USA) for 2 h. The fixative was aspirated and cleaned with DPBS and post-fixed in 1% osmium tetroxide for 30 min. After fixation, samples were dehydrated in a graded series of ethanol solutions. After critical point drying, the specimens were sputter-coated with Pt and microscopically examined using field-emission scanning electron microscopy (FE-SEM, Apreo S, Thermo Fisher Scientific, MA, USA) at 10 kV. The colonies were counted as colony-forming units per milliliter (CFU/mL), and the morphological changes in the fungal cell colonies on the specimen surfaces were examined.

To measure the flexural strength of the denture base materials in accordance with ISO 20795-1:2013 [41], the bar-shaped 3DP denture resin specimens (n = 10) subjected to different PPTs with and without microcapsules were prepared and stored in 37 °C distilled water for 50 h. The flexural strength of each bar-shaped specimen was measured using a three-point bending test apparatus with a universal testing machine (Instron 8871; Instron, Canton, MA, USA) of 5kN load cell at a crosshead speed of 5 mm/min. The flexural strength (FS) was computed using the following equation:

$${\text{FS}} = \frac{{3Pl}}{{2bd^{2} }},$$

where *P* is the maximum load before fracture, *l* is the distance between the supports (50 mm), *b* is the specimen width, and *d* is the specimen thickness. After testing, the fractured surfaces were sputter-coated with Pt (5-nm layer thickness) and observed using FE-SEM for fractographic analysis.

A Fourier transform infrared spectrometer (FT-IR, TENSOR27, Bruker, Germany) and data collection program (OPUS, OPtical User Software, Bruker, Germany) was used to investigate the effect of PPT on the degree of conversion (DC) of the 3DP denture resin specimens with microcapsules. Considering the PPT period, the infrared spectra of 3DP denture resin discs containing phytoncide-filled microcapsules, as well as those without microcapsules, were recorded between 4000 cm^− 1^ and 400 cm^− 1^ in the absorbance mode (n = 3 per group), with a resolution of 1 cm^− 1^. The FTIR spectra of unpolymerized denture base resins were also recorded. DC of each 3DP denture resin specimen was calculated using the following equation.

$${\text{Degree}}\;{\text{of}}\;{\text{Conversion}}\left( {{\text{DC}}} \right) = \left\{ {1 - \frac{{Abs_{{aliphatic}} ~\left( {polymer} \right)/Abs_{{aromatic}} ~\left( {polymer} \right)}}{{Abs_{{aliphatic}} ~\left( {monomer} \right)/Abs_{{aromatic}} ~\left( {monomer} \right)}}} \right\} \times 100$$

where *Abs*_*aliphatic*_ is the absorbance peak intensity of the aliphatic bond of the 3DP denture resin material measured from the peak at 1638 cm^− 1^ and *Abs*_*aromatic*_ is the absorbance peak intensity of the aromatic bond of the 3DP denture resin material measured from the peak at 1610 cm^− 1^.

To investigate the hardness of the 3DP denture resin disc, the discs with and without phytoncide microcapsules, subjected to different PPT protocols (n = 3 per group), were prepared. Minimizing the effect of microcapsules on the surface topography, the testing surface of each disc was polished for clear examination of indentation using a polishing machine (Phoenix Beta, Bühler, Düsseldorf, Germany) at 150 rpm with 4000 grit SiC papers. The Vickers hardness test was performed on the polished surface of each disc using a diamond pyramid indenter (HM-220B, Mitutoyo, Japan) at 9.8 N load and repeated 10 times for each disc-shaped specimen.

The 3DP denture base-shaped specimens with and without microcapsules were subjected to different PPT protocols (n = 10 per group), and their intaglio surfaces were digitized using a high-resolution laboratory scanner (Medit T710; Medit Corp, Seoul, Korea) in standard operation mode. The intaglio surface trueness of each specimen was evaluated using a 3D inspection software (Geomagic Control X; 3D Systems, Rock Hill, SC, USA) by superimposing the intaglio area of the reference CAD data of the denture base and the scanned intaglio surface data of the specimens. Three pairs of corresponding points were carefully selected on the tissue surface of each scanned denture base and reference CAD data to achieve preliminary alignment. Subsequently, the best-fit alignment with the iterative closest point-matching algorithm was performed. A color-coded 3D deviation map was displayed for each superimposition analysis of the 3DP specimens. The nominal deviation was set to ± 50 μm, and the critical deviation was set to ± 500 μm. The area within nominal deviation was displayed in green, while the area greater than nominal deviation was displayed in yellow (positive) or blue (negative). The surface deviation data measured in root-mean-square estimates (RMS, µm) were calculated using the formula below:

$${\text{RMS}} = \frac{{\sqrt {\mathop \sum \nolimits_{{i = 1}}^{n} \left( {X_{{1,i}} - X_{{2,i}} } \right)^{2} } }}{{\sqrt n }}$$

where *X*_*1,i*_ is the point *i* on the reference data; *X*_*2,i*_ is the point *i* on the test data; *n* is the total number of points.

The statistical analyses were conducted using the measured data of the 3DP denture resin specimens from five different treatment categories: (1) no microcapsule and 5-min PPT (negative control), (2) microcapsule and 5-min PPT (positive control), (3) microcapsule and 10-min PPT, (4) microcapsule and 20-min PPT, and (5) microcapsule and 30-min PPT. Based on the distribution of the collected data tested by the Shapiro–Wilk’s method and Levene’s test of equal variance, a non-parametric Kruskal–Wallis test and a post-hoc multiple comparison with Wilcoxon rank test adjusted by Bonferroni’s method were conducted for the data collected from the antifungal activity and degree of conversion analyses. For the measurements of each test for flexural strength (MPa), Vickers hardness (VH), and intaglio surface trueness (RMS, µm), one-way analysis of variance (ANOVA) and a post-hoc multiple comparison using Tukey’s method were performed for the differences among the groups. All statistical analyses were performed using IBM SPSS Statistics, v25.0; IBM Corp., Armonk, NY, USA), with a level of statistical significance (α) of 0.05.

## Results

Based on the Kruskal-Wallis test, the numbers of fungal cell colonies (CFU/mL) on the discs with microencapsulated phytoncide were significantly different between the groups (Fig. 1, *P* < 0.001). Adherence of fungal cell colonies to the disc surface was the lowest in the specimens with microcapsules and 10-min PPT, which indicates the highest antifungal activity. The specimens with microcapsules and 5-min PPT (positive control) showed significantly higher antifungal activity than those without microcapsules and 5-min PPT (negative control, *P* < 0.05). However, as the PPT increases from 10 to 30 min, the numbers of fungal cell colonies attached to the discs filled with microcapsules significantly increased (all *P* < 0.05).

**Fig. 2:**
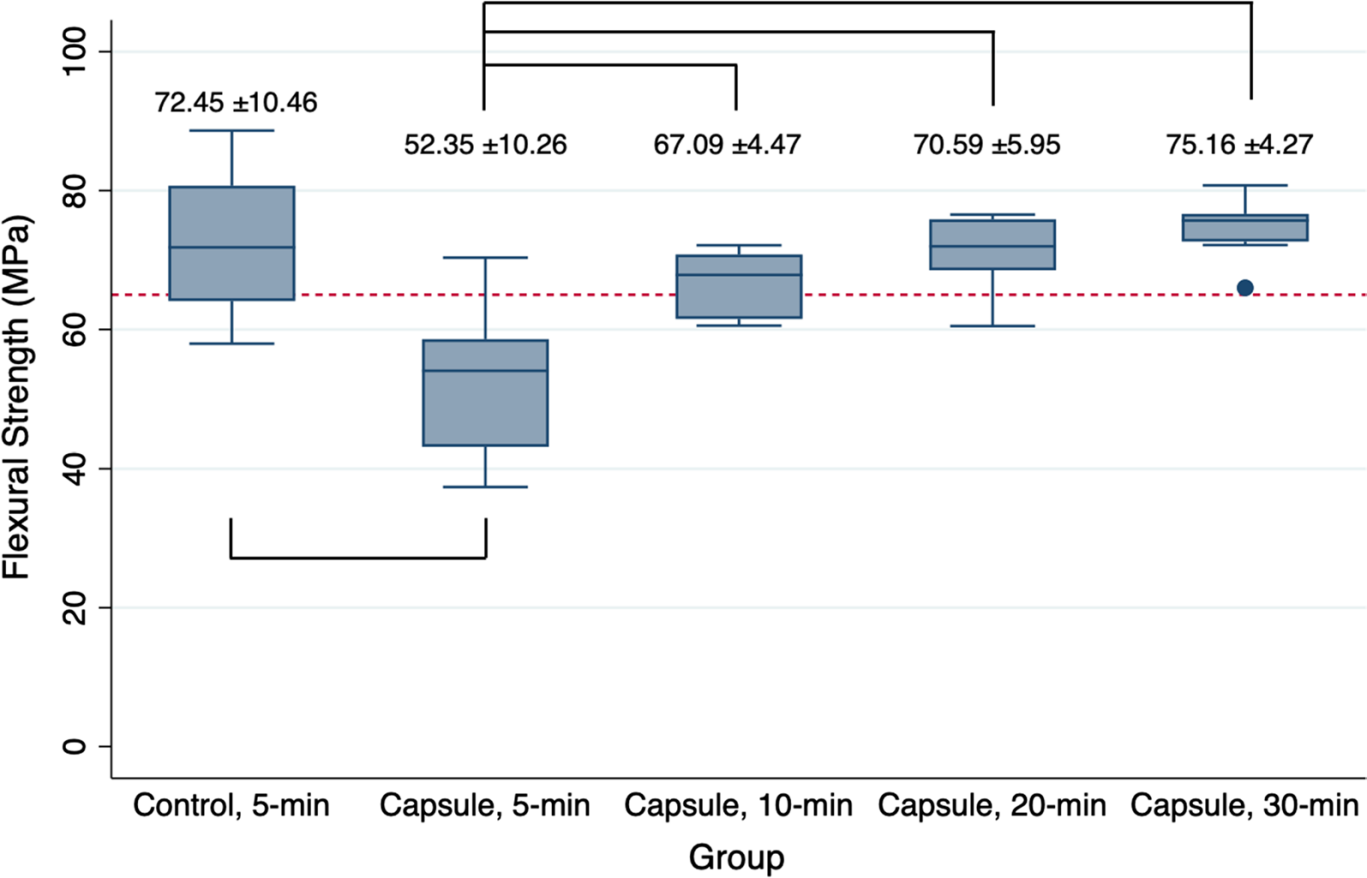
Flexural strength values (MPa) of bar-shaped 3D-printed denture base resin specimens subjected to different post-polymerization time (PPT) protocols: 5-min, 10-min, 20-min, and 30-min. Control: 3D-printed denture base resin specimens without microcapsules; Capsule: 3D-printed denture base resin specimens with 5 wt% phytoncide-filled microcapsules. Significant differences between the groups are marked with black lines (*P* < 0.05). Flexural strength value of 65 MPa is marked in red dotted line

**Fig. 3:**
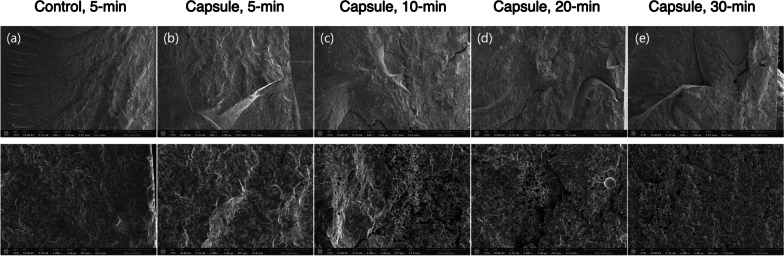
Representative microscopic images (upper row, ×200, and lower row, ×2000) of fractured 3D-printed bar-shaped denture base specimens subjected to different post-polymerization time (PPT) protocols: 5-min, 10-min, 20-min, and 30-min. Control: 3D-printed denture base resin discs without microcapsules; Capsule: 3D-printed denture base resin discs with 5 wt% phytoncide-filled microcapsules. **a** Brittle fracture with smooth surface and chevron marks; **b**–**e** intermediate (ductile) fracture with rough surface and jagged areas. Note the microcapsules are evenly distributed over the fractured surfaces

Figure 2 shows the mean flexural strength values of the specimens subjected to different PPT protocols. The mean strength value of the specimens with no microcapsules and 5-min PPT (negative control) was 72.45 ± 10.46 MPa. With the same PPT, incorporating microcapsules into the denture base resin significantly decreased the mean value to 52.35 ± 10.26 MPa (positive control, *P* < 0.05). However, when subjected to 10-min PPT, the mean strength value of the specimens with microcapsules significantly increased up to 67.09 ± 4.47 MPa (*P* < 0.05). As the PPT increased to 20 and 30 min, the specimens with microcapsules showed significantly improved mean strength values compared with those from the positive control group (all, *P* < 0.05). The specimen with microcapsules and treated with 10-min PPT revealed comparable strength as those without microcapsules (negative control, *P* > 0.05). Microscopic observations (Fig. 3) revealed that the specimens from the negative control group showed brittle fracture patterns with flat, compact, and organized surfaces. However, irrespective of the PPT protocols, all specimens with microcapsules showed typical patterns of intermediate fractures.

**Fig. 4:**
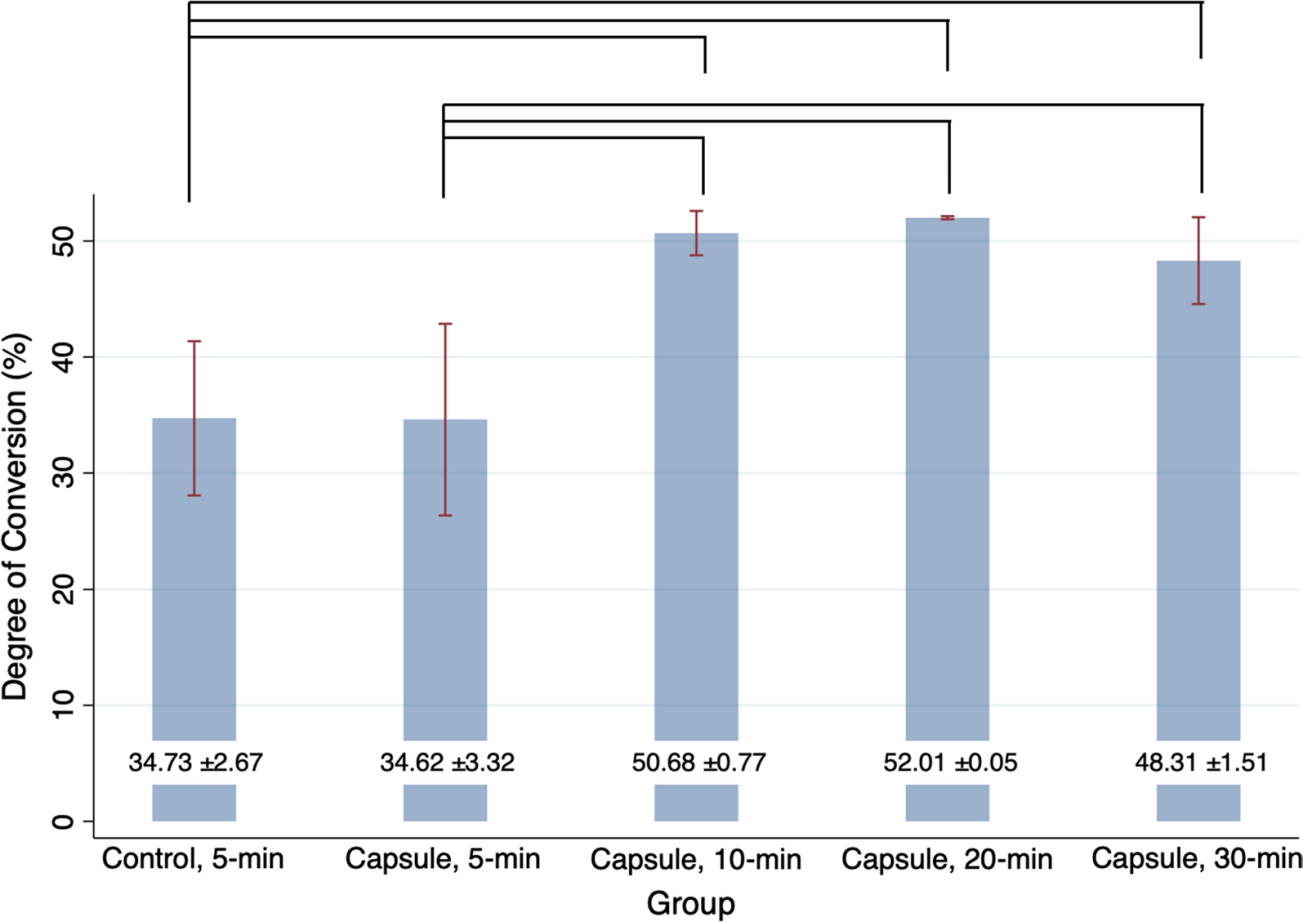
Degree of conversion (%) of 3D-printed denture base resin discs subjected to different post-polymerization time (PPT) protocols: 5-min, 10-min, 20-min, and 30-min. Control: 3D-printed denture base resin discs without microcapsules; Capsule: 3D-printed denture base resin discs with 5 wt% phytoncide-filled microcapsules. Significant differences between the groups are marked with black lines (*P* < 0.05)

The DC values of the discs with microencapsulated phytoncide were significantly different between the groups, according to the Kruskal-Wallis analysis (*P* = 0.014). The 3DP denture resin discs filled with microcapsules and subjected to PPT for no less than 10 min showed significantly improved DC compared with those subjected to 5-min PPT (*P* < 0.05, Fig. 4). From the highest to lowest of the DC values, the discs subjected to 20-min PPT (52.01 ± 0.05%) were followed by those subjected to 10-min PPT (50.68 ± 0.77%), 30-min PPT (48.31 ± 1.51%), and 5-min PPT (34.62 ± 3.32%, positive control). No difference was found between the DC values of the specimens from the positive and negative control groups (*P* > 0.05). In addition, there was no statistical difference in the DC values between the specimens treated with 10-, 20-, and 30-min PPTs (all, *P* > 0.05).

**Fig. 5:**
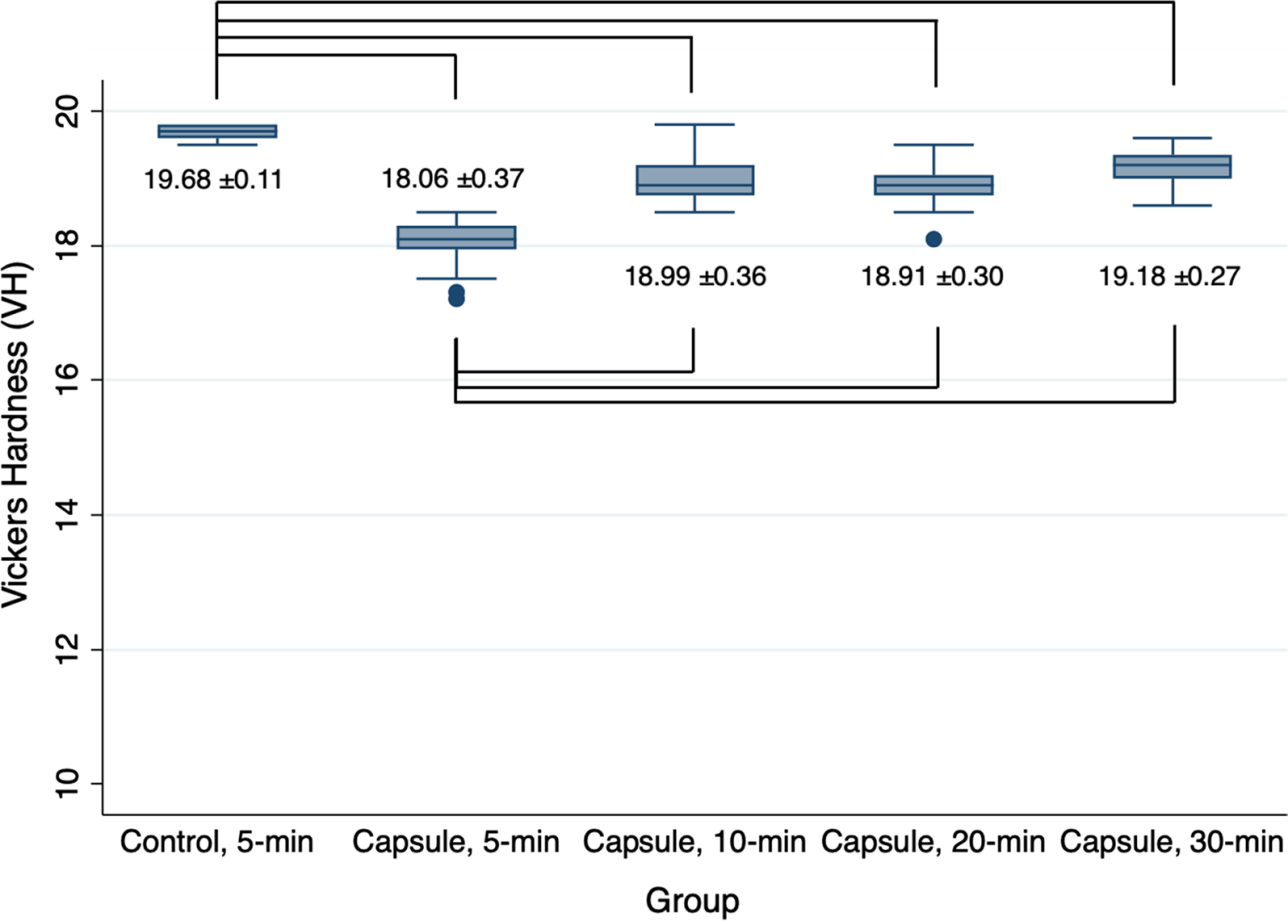
Vickers hardness (VH) of 3D-printed denture base resin discs subjected to different post-polymerization time (PPT) protocols: 5-min, 10-min, 20-min, and 30-min. Control: 3D-printed denture base resin discs without microcapsules; Capsule: 3D-printed denture base resin discs with 5 wt% phytoncide-filled microcapsules. Significant differences between the groups are marked with black lines (*P* < 0.05)

The mean hardness value (HV) of the specimens without microcapsules and subjected to 5-min PPT (negative control) was significantly higher than that of those with microcapsules, regardless of the changes in PPT protocols (all, *P* < 0.05, Fig. 5). The mean hardness value of the specimens from the positive control group (18.06 ± 0.37) was the lowest among the tested groups. However, on using PPT protocols of no less than 10 min, the mean hardness values of the specimens with microcapsules significantly increased compared with that of those subjected to 5-min PPT (all, *P* < 0.05). The mean hardness value was 18.99 ± 0.36 for the specimens subjected to 10-min PPT. No difference was detected between the groups filled with microcapsules and subjected to 10-min, 20-min, and 30-min PPT (all, *P* > 0.05).

**Fig. 6:**
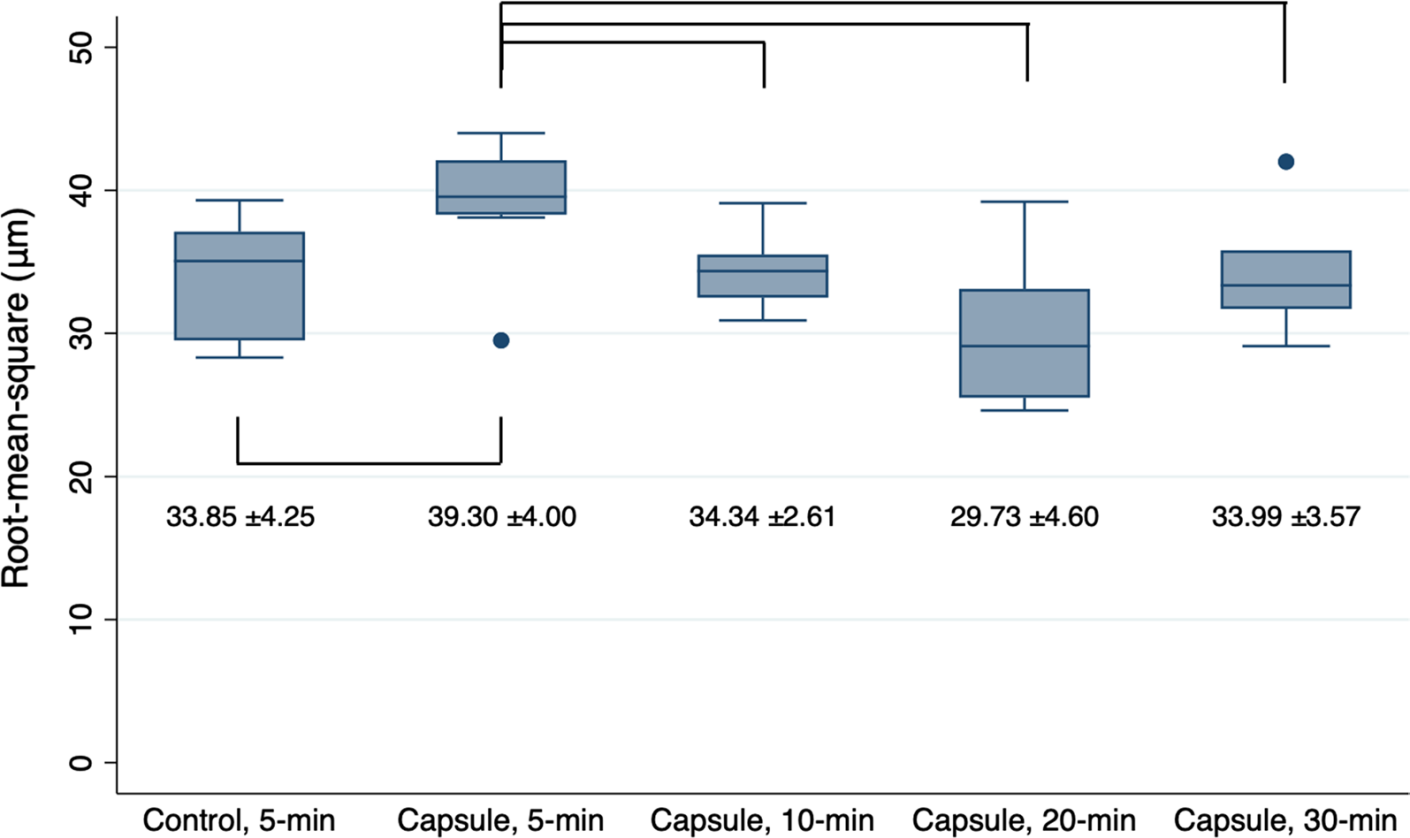
Root-mean-square estimates of the 3D-printed denture base resin discs subjected to different post-polymerization time (PPT) protocols: 5-min, 10-min, 20-min, and 30-min. Control: 3D-printed denture base resin discs without microcapsules; Capsule: 3D-printed denture base resin discs with 5 wt% phytoncide-filled microcapsules. Significant differences between the groups are marked with black lines (*P* < 0.05)

**Fig. 7:**
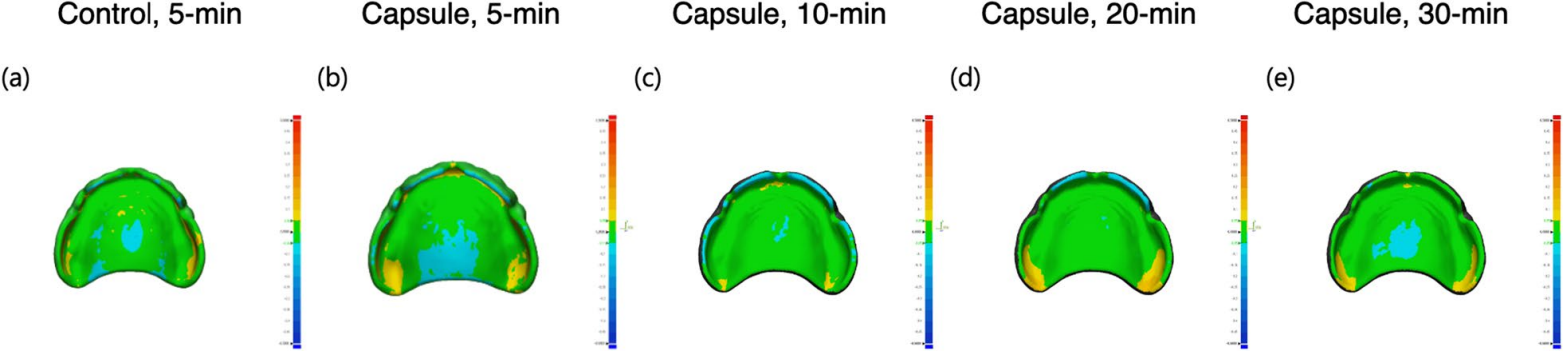
Representative images of color deviation maps of the 3D-printed denture base resin discs subjected to different post-polymerization time (PPT) protocols: 5-min, 10-min, 20-min, and 30-min. Control: 3D-printed denture base resin discs without microcapsules; Capsule: 3D-printed denture base resin discs with 5 wt% phytoncide-filled microcapsules. **a** Control group, with 5-min PPT, **b** Capsule group, with 5-min PPT, **c** Capsule group, with 10-min PPT, **d** Capsule group, with 20-min PPT, and **e** Capsule group, with 30-min PPT.

The highest mean RMS value was 39.30 ± 4.00 μm for the specimens with microcapsules and subjected to 5-min PPT. In contrast, the lowest value was 29.73 ± 4.60 μm for those subjected to 20-min PPT. Under the same PPT of 5 min, the specimens with microcapsules showed significantly inferior trueness of the intaglio surface compared with that of those without microcapsules (*P* < 0.05, Fig. 6). As the PPT increased, all specimens with microcapsules and subjected to PPT for no less than 10 min showed better trueness than those subjected to 5-min PPT (positive control, *P* < 0.05). No statistical differences were detected between the specimens with microcapsules and subjected to 10-min, 20-min, and 30-min PPT protocols (all, *P* > 0.05). Furthermore, the specimens filled with microcapsules and treated with PPT protocols for no less than 10 min were comparable with that of those without microcapsules (negative control, *P* > 0.05). However, regardless of the inclusion of microcapsules or the PPT protocol, all the specimens showed the RMS values under 50 μm for overall intaglio surfaces (Figs. 6 and 7). The color deviation map (Fig. 7) revealed that the specimens with microcapsules and subjected to 10-min PPT showed the most favorable pattern of surface deviation, with overall green area (within 50 μm of nominal deviation) with small regions of positive (yellow) or negative (blue) deviations.

## Discussion

A group of researchers previously reported the fabrication of denture base material with antifungal activity against *C. albicans* using microencapsulation of phytoncide oil extract, mixing with a proper dispersant, and a digital light processing technique [31]. However, adding phytoncide-filled microcapsules may have affected the mechanical properties and dimensional accuracy of the 3DP denture base [37]. Although the flexural strength of the 3DP denture bases with microcapsules was comparable with that of bases without microcapsules, the degree of conversion, hardness, or dimensional accuracy was relatively decreased [31, 37]. To improve the flexural strength, degree of conversion, hardness, and intaglio surface trueness while maintaining the antifungal effect of the 3DP denture base, the current study focused on the effect of PPT, one of the most critical phases for 3D printing. Based on the findings of this study, the null hypotheses were rejected.

Flexural strength is essential for the success of denture treatment, as an external loading force is exerted on the dentures during mastication [43, 44]. The flexural strength of 3DP denture resin was enhanced by increasing the PPT [40]. In this study, compared with 5-min PPT, 3DP denture bases with phytoncide-filled microcapsules showed improved flexural strength after increasing the PPT to 10 min. The measured strength was higher than 65 MPa, which is necessary for base polymers to be clinically acceptable based on the recommendations of the International Standard Organization [41]. This may be in accordance with the significantly increased DC in the specimens with microcapsules subjected to 10-min PPT compared with that of those subjected to 5-min PPT. Generally, the pores may act as the point of crack initiation during the fracture of PMMA resin; the microcapsules incorporated in the 3DP denture base resin may also function similarly [37]. In this study, the increased PPT was beneficial in enhancing the degree of conversion and, consequently, the flexural strength. Insufficient DC could reduce the mechanical properties of denture resins, where high DC is related to high conversion from monomers to polymers [45]. Furthermore, a denture base must have adequate hardness to resist surface wear during mastication or maintenance care, which may affect microbial accumulation when using complete dentures [46, 47]. The addition of microcapsules may have affected the Vickers hardness of 3DP denture base resin, still higher than the value reported in the literature [48]. In fact, by increasing PPT no less than 10 min, 3DP denture base resin with antifungal microcapsules showed sufficient Vickers hardness to withstand the clinical conditions [48].

For DLP-based workflow, a light source is essential as it supplies sufficient energy to induce polymerization of the photosensitive resin material, resulting in the formation of a highly cross-linked polymer [49]. Furthermore, a post-polymerization process is required to fully polymerize the object that has just been 3DP to finalize the desired mechanical and surface properties. Among the several parameters that could be modified, PPT is one of the easiest factors to approach. By changing the PPT, the accuracy, flexural strength, and hardness of the 3DP prosthesis have been improved, suggesting that the increase in PPT is advantageous [38–40]. In this study, by changing PPT from 5 min (positive control) to 10 min, the antifungal activity and mechanical properties of the 3DP denture base containing microcapsules were significantly improved. However, PPT protocols longer than 10 min did not increase the antifungal effect of the 3DP denture base. In addition, the intaglio surface trueness of the 3DP denture base with microcapsules, which is paramount for intimate tissue surface adaptation of the prosthesis, was also evaluated in this study. With the 10-min PPT protocol, the 3DP denture bases with microcapsules showed intaglio surface trueness comparable with that of those without.

The experimental design and calculated sample size evaluated in the present study were referred to previous in-vitro research by Jeon et al. and other recent in-vitro studies with similar research designs and test materials that reported significant differences [31, 37, 40]. However, the absence of a priori power analysis could be considered as a limitation of the this in-vitro research. In addition, further in-vitro research regarding physico-mechanical properties such as fracture toughness, compressive strength, and color stability of 3DP denture base with microcapsules is required. Since only one of the numerous 3D-printable denture base materials was tested in this study, further experiments with other denture materials are required.

## Conclusion

Within the limitations of this in vitro study, the 3DP denture base containing phytoncide-filled microcapsules at 5 wt% concentration and subjected to 10-min PPT showed sufficient antifungal activity, while maintaining clinically comparable flexural strength, Vickers hardness, and intaglio surface trueness to the original 3DP denture base resin with no microcapsules. The antifungal activity and degree of conversion of 3DP denture base resin with microcapsules were significantly affected by the change of PPT. Further in-vitro and in-vivo studies are required before applying for clinical purpose.

## Data Availability

The datasets generated and/or analyzed during the current study are available from the corresponding author upon reasonable request.
